# Genetic diversity of *Giardia* isolates from patients in Chandigarh region: India

**DOI:** 10.1186/s13104-020-05419-1

**Published:** 2021-01-19

**Authors:** Shabnam Thakur, Upninder Kaur, Rakesh Sehgal

**Affiliations:** Department of Medical Parasitology, Postgraduate Institute of Medical Research and Education, Chandigarh, 160012 India

**Keywords:** *Giardia*, Genotype, *GLUD1* (glutamate dehydrogenase), PCR, Sequencing

## Abstract

**Objective:**

The aim of study was to characterize *Giardia* isolates genetically among patients in Chandigarh region, India. For this, nested PCR targeting fragment of the glutamate dehydrogenase (*GLUD1* earlier named as GDH) gene was used. Phylogenetic analysis was done by constructing neighbor-joining tree made out of the nucleotide sequences of *G*. *intestinalis* isolates obtained in this study and with the known sequences published in GenBank.

**Results:**

Out of 40 samples, *GLUD1* gene was amplified in 33 samples (82.5%). The product of *GLUD1* gene was successfully sequenced only in 32 samples. In these samples, assemblage B was found in 27 (84.37%) samples whereas 5 (15.6%) samples had assemblage A. Among assemblage B most of them were of BIII. Therefore, genotyping of *Giardia* would be helpful in conducting epidemiological studies.

## Introduction

*Giardia intestinalis* is well known intestinal parasite of humans and mammals. *Giardia* causes approximately 280 million cases of giardiasis worldwide annually [1]. Most of these cases are associated with lower socioeconomic status. In the year 2004, giardiasis was included in WHO ‘Neglected Diseases Initiative’ because of its high prevalence in communities with low socio-economic status.

Giardiasis is acquired due to ingestion of cysts of *Giardia* in water or food [2]. *Giardia intestinalis* is composed of eight major genotypes or assemblages (A–H) [2]. Genotype A and B are common among humans having variable distribution frequency in different geographical locations and these assemblages mainly considered as zoonotic assemblages as they are able to infect both men and animals [3]. These assemblages are further divided into subassemblages on the basis of either digestion by restriction enzyme or sequence analysis. Assemblages A are classified as AI, AII,AIII and AIV. Subassemblages AI and AII were commonly found in humans while AI, AIII and AIV are subassemblages of animals. Zoonotic potential is linked with only subassemblage AI [4]. Assemblage B, catogorised into four sub-assemblages BI, BII, BIII and BIV. As per literature subassemblages BIII and BIV were reported in humans while other two are specific for animals [4, 5]. The BIII sub-assemblage is closer to sub-assemblages BI and BII and therefore has zoonotic potential. The *GLUD1* (earlier known as GDH) locus has been utilized for genetic characterization of *G. intestinalis* isolates in vertebrates [4] hosts and is able to categorize them into sub genotypes/subassemblages. The present work was aimed to determine assemblages and sub-assemblages of *Giardia* isolates involved in its transmission by using glutamate dehydrogenase (*GLUD1*) marker.

## Main text

### Materials and methods

#### Sample collection

Forty microscopic *Giardia* positive stool samples were collected from the Routine Laboratory of Department of Medical Parasitology, PGIMER, Chandigarh from August 2019 to December 2019.

#### DNA extraction

From stool samples, DNA was extracted by using QIAmp Fast DNA Stool Mini Kit (QIAGEN, Germany) as per manufacturer’s instructions with slight modifications. The suspension was initially incubated at 90 °C for 15 min and then for another 30 min at 75 °C. DNA was eluted in 50 µl of AE buffer. DNA concentration was measured by NanoQuant (Infinite® 200 PRO NanoQuant) and stored at − 20 °C until further use.

#### Polymerase chain reaction amplification

The two-step PCR was employed for the amplification of  *GLUD1* gene (432 bp) by using previously published primers given by Read et al. [6]. The conditions and primers for both primary and secondary reactions are given as Additional file 1: Table S1. The first set of PCR reaction comprised of 2.0 μL of DNA template, 12.5 μL 2 × Go Taq Green Mix, 1 μL of each primer (10 μM), 1 μL of Bovine Serum Albumin (BSA) whereas in case of secondary PCR, DNA template was replaced by the product of primary reaction. For the negative control, nuclease-free water and for the positive control, DNA of *Giardia* strain, Portland 1 was used for each PCR reaction. All the precautions were taken to prevent contamination.

#### PCR product purification and sequencing

**Fig. 1:**
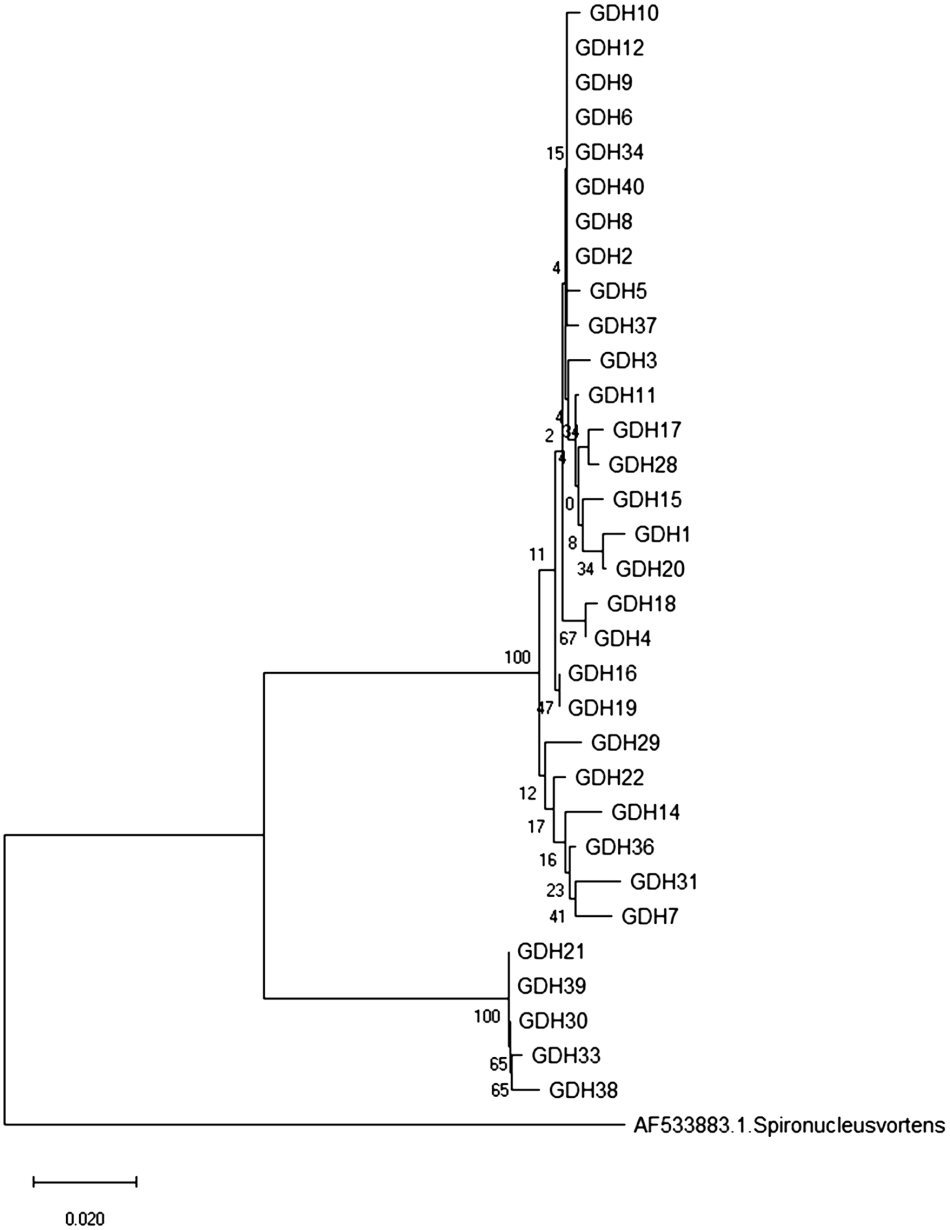
Phylogenetic tree constructed with the neighbor-joining method using nucleotide sequences of *GLUD1* gene. The sequence of *Spironucleus vortens* was used as an out-group

All PCR-positive samples were sequenced using secondary primers. PCR products were sequenced in both forward and reverse directions. By using BLAST, nucleotide similarity of sequenced amplicons was searched in GenBank (http://www.ncbi.nlm.nih.gov/blast). CLUSTAL X was used to determine multiple sequence alignments. Neighbor-joining distance trees were prepared using MEGAX software (https://www.megasoftware.net/) (Fig. 1). Bootstrap values were based on 1000 replicas. All the sequences obtained during the study were submitted to the GenBank (Accession number: MT584168–MT584199).

The direct links which are publicly available are as follows:

https://www.ncbi.nlm.nih.gov/nuccore/MT584168

https://www.ncbi.nlm.nih.gov/nuccore/MT584169

https://www.ncbi.nlm.nih.gov/nuccore/MT584170

https://www.ncbi.nlm.nih.gov/nuccore/MT584171

https://www.ncbi.nlm.nih.gov/nuccore/MT584172

https://www.ncbi.nlm.nih.gov/nuccore/MT584173

https://www.ncbi.nlm.nih.gov/nuccore/MT584174

https://www.ncbi.nlm.nih.gov/nuccore/MT584175

https://www.ncbi.nlm.nih.gov/nuccore/MT584176

https://www.ncbi.nlm.nih.gov/nuccore/MT584177

https://www.ncbi.nlm.nih.gov/nuccore/MT584178

https://www.ncbi.nlm.nih.gov/nuccore/MT584179

https://www.ncbi.nlm.nih.gov/nuccore/MT584180

https://www.ncbi.nlm.nih.gov/nuccore/MT584181

https://www.ncbi.nlm.nih.gov/nuccore/MT584182

https://www.ncbi.nlm.nih.gov/nuccore/MT584183

https://www.ncbi.nlm.nih.gov/nuccore/MT584184

https://www.ncbi.nlm.nih.gov/nuccore/MT584185

https://www.ncbi.nlm.nih.gov/nuccore/MT584186

https://www.ncbi.nlm.nih.gov/nuccore/MT584187

https://www.ncbi.nlm.nih.gov/nuccore/MT584188

https://www.ncbi.nlm.nih.gov/nuccore/MT584189

https://www.ncbi.nlm.nih.gov/nuccore/MT584190

https://www.ncbi.nlm.nih.gov/nuccore/MT584191

https://www.ncbi.nlm.nih.gov/nuccore/MT584192

https://www.ncbi.nlm.nih.gov/nuccore/MT584193

https://www.ncbi.nlm.nih.gov/nuccore/MT584194

https://www.ncbi.nlm.nih.gov/nuccore/MT584195

https://www.ncbi.nlm.nih.gov/nuccore/MT584196

https://www.ncbi.nlm.nih.gov/nuccore/MT584197

https://www.ncbi.nlm.nih.gov/nuccore/MT584198

https://www.ncbi.nlm.nih.gov/nuccore/MT584199

### Results

**Fig. 2:**
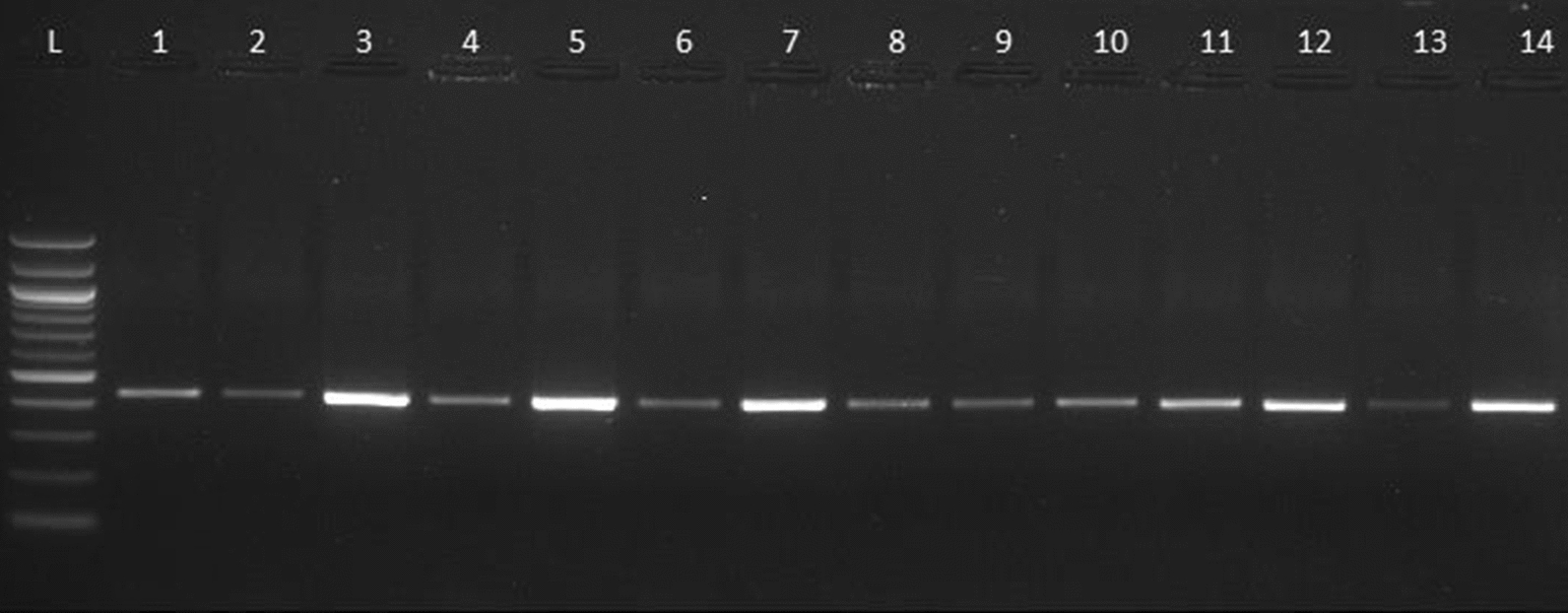
Nested-PCR amplification of *Giardia intestinalis* *GLUD1* gene (432bp) 1.5% agarose gel stained with Ethidium bromide. L, ladder (100bp); lane1–13, *Giardi*a positive samples; lane14, positive control

Out of 40 samples, the *GLUD1* gene was amplified in 33 samples (82.5%) (Table1, Fig. 2). The possible reason for this occurrence indicated the less parasitic load in these isolated samples. The product of *GLUD1* gene was successfully sequenced only in 32 samples. In these samples, assemblage B was found in 27 (84.37%) among which 18 (66.66%) were sub-genotype BIII and 9 (33.33%) were sub-genotype BIV samples whereas 5 (15.6%) samples had assemblage A. Four of them were AI subgenotype and only1 belonged to sub-genotype AII.

**Table 1:** PCR results of samples with their assemblages

Sample No	PCR	Assemblages
1	+ve	B
2	+ve	B
3	+ve	B
4	+ve	B
5	+ve	B
6	+ve	B
7	+ve	B
8	+ve	B
9	+ve	B
10	+ve	B
11	+ve	B
12	+ve	B
13	+ve	Unable to sequence
14	+ve	B
15	+ve	B
16	+ve	B
17	+ve	B
18	+ve	B
19	+ve	B
20	+ve	B
21	+ve	A
22	+ve	B
23	−ve	–
24	−ve	–
25	−ve	–
26	−ve	–
27	−ve	–
28	+ve	B
29	+ve	B
30	+ve	A
31	+ve	B
32	−ve	–
33	+ve	A
34	+ve	B
35	−ve	–
36	+ve	B
37	+ve	B
38	+ve	A
39	+ve	A
40	+ve	B

### Discussion

*Giardia intestinalis* is the most common and frequent intestinal parasitic agent of gastroenteritis mainly in the developing countries [7]. The present study provides data on genetic diversity of *Giardia* isolates from patients in the Chandigarh region. As per results, assemblage A and B are common among this population, which is in concordance to other studies [8, 9]. In our study, assemblage B was the predominant genotype observed followed by assemblage A. However, similar observations were previously reported and observed worldwide which showed that assemblage B was predominant in comparison to assemblage A [9–12]. There are studies which showed the presence of assemblage B in Rhesus macaques (*Macaca mulatta*) and potable water resources of Northern India [13, 14]. But other studies have reported the assemblage A as the predominant genotype in other regions [15]. Due to geographical variations, differences were observed in the prevalence of various genotypes and the detection of these variations would be helpful in designing effective therapeutic approaches*.*

### Conclusion

The results showed that PCR sequencing and phylogenetic analysis is an excellent molecular technique for genotyping of *Giardia intestinalis*. Detection of *Giardia intestinalis* assemblages and sub-assemblageswould be helpful in conducting epidemiological studies.

## Limitation of study

Present study involves only single locus for genotyping and also the sample size is less so it is difficult to interpret zoonotic potential of these isolates. Therefore, multi-locus typing data is required to differentiate between *Giardia* isolates.

## Supplementary Information

**Additional file 1: Table S1.** Sequence of primers and sgRNA were listed.

## Data Availability

All the supporting data related to the present work is available with the authors and the sequences obtained during the study were submitted to the GenBank (Accession number: MT584168–MT584199).

## Abbreviations

BLAST: Basic Local Alignment Search Tool
DNA: Deoxyribonucleic Acid
PCR: Polymerase Chain Reaction
BSA: Bovine Serum Albumin
