# Supplementary material for: Genetic diversity of Giardia isolates from patients in Chandigarh region: India
Source: BMC Res Notes. 2021 Jan 19;14:26. doi: 10.1186/s13104-020-05419-1 (PMC7816347; doi:10.1186/s13104-020-05419-1)
Supplement: Supplementary file 1 — Additional file 1: Table S1. Sequence of primers and sgRNA were listed. [file 13104_2020_5419_MOESM1_ESM.docx]

**Table S1: PCR conditions and primers**

| **Gene** | **Primer name** | **Primer sequence** | **Denaturation** | **Anealing** | **Elongation** | **No of cycles** |
| --- | --- | --- | --- | --- | --- | --- |
| **gdh- Primary** | GDHeF | TCAACGTYAAYCGYGGYTTCCGT | 94°C , 30 s | 54°C, 30 s | 72°C, 45s | 40 |
|  | GDHiR | GTTRTCCTTGCACATCTCC |  |  |  |  |
| **gdh- secondary** | GDHiF | CAGTACAACTCYGCTCTCGG | 94°C , 45 s | 54°C, 45 s | 72°C, 30s | 40 |
|  | GDHiR | GTTRTCCTTGCACATCTCC |  |  |  |  |
